# Amelogenesis imperfecta: A challenge to restoring esthetics and function

**DOI:** 10.4103/0972-124X.75917

**Published:** 2010

**Authors:** V. Ranganath, Ashish S. Nichani, V. Soumya

**Affiliations:** *Department of Periodontics, AECS Maaruti College of Dental Sciences and Research Centre, Bangalore, Karnataka, India*

**Keywords:** Anterior dental esthetics, crown-lengthening, interdisciplinary dentistry, occlusion

## Abstract

Rehabilitation of complicated cases poses difficulty in clinical practice, both with respect to restoring function and with esthetics. One such clinical condition where the dentist has to give importance to proper planning of the treatment and execution of the plan is amelogenesis imperfecta (AI), a condition where both function and esthetics are accommodated. This article discusses both the functional and esthetic rehabilitation of a patient with AI. Both the esthetics and function were hampered in this patient due to the condition. As a result, the treatment was properly planned and executed. A number of treatment options are available for us today to treat such a case. There is no one technique to be followed as such. However, the aim was to properly diagnose the case and provide good function and esthetics to the patient.

## INTRODUCTION

Amelogenesis imperfecta (AI) is a clinical condition, which comprises developmental disorders that demonstrate alterations in the enamel. It represents a group of inherited disorders, which are clinically heterogeneous and exhibit tooth enamel defects in the absence of systemic manifestations.[1] Both primary and permanent dentitions are affected.[2] The predominant clinical manifestations of affected individuals are enamel hypoplasia (enamel is seemingly correctly mineralized but thin), hypomineralization (subdivided into hypomaturation and hypocalcification), or a combined phenotype, which is seen in most cases.[3] The trait of AI can be transmitted by an autosomal dominant, autosomal recessive, or X-linked mode of inheritance.[4,5] The distribution of AI types is known to vary among different populations. In a study in Sweden, 63% of the cases were inherited as autosomal dominant.[3] In contrast, in a study in the Middle East, the most common prevalent type of AI was found to be autosomal recessive.[6,7]

There are a number of symptoms, which include the following.

- Thin tooth enamel
- Soft tooth enamel
- Pitted tooth enamel
- Rough tooth enamel
- The teeth are easily damaged
- Teeth appear yellow

Also, the radiographic examination usually shows a full complement of teeth, but the crowns of the teeth have very thin enamel or lack enamel completely.

## CASE REPORT

**Figure 1:**
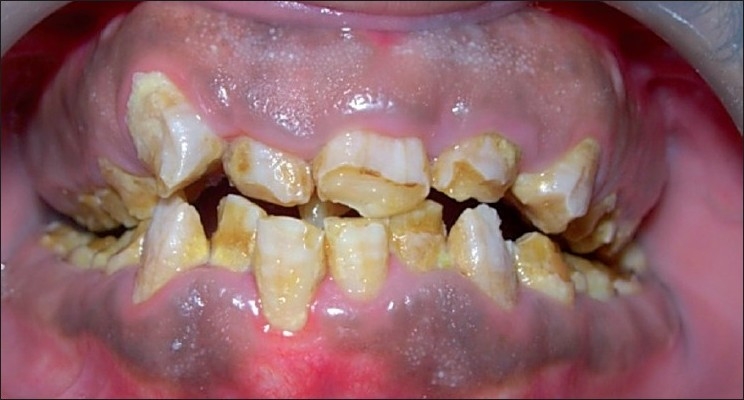
Clinical appearance of amelogenesis imperfecta. Labial view of maxillary and mandibular teeth

A 22-year-old male patient presented with the chief complaint of considerable sensitivity of his teeth, difficulty in chewing, and displeasure with his present dental appearance. A detailed medical, dental, and social history was obtained. The enamel of all the teeth was hypoplastic and yellow brown in color. The surfaces of the teeth were rough, and the enamel was either not visible or very thin over the crowns of all the teeth. The dentin, where it was exposed, was brown [Figure 1]. Examination revealed a midline discrepancy, due to missing 21. A mesiodens was present between 11 and 21. There was definite loss of vertical dimension because the molars were highly demineralized. The diagnosis of AI was made through the extraction of the mesiodens, which was later kept under observation for the procedure of decalcification in the Department of Oral Pathology, AECS Maaruti College of Dental Sciences and Research Centre, Bangalore.

The gingival status of the patient was evaluated and we noticed a thick gingival biotype. Pseudoperiodontal pockets were also present.

Complete maxillary and mandibular impressions were made and diagnostic casts were obtained.

**Figure 2:**
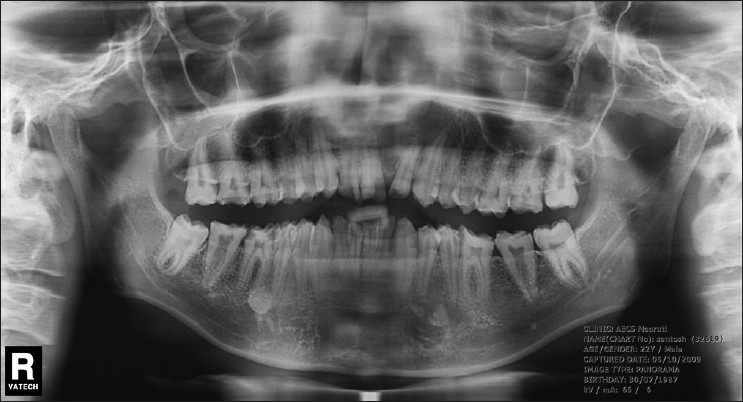
Panoramic radiograph. Radiodensities of enamel and dentin were similar

Preoperative intraoral photographs and radiographs were also obtained [Figure 2].

Once these were done, the complete treatment plan was explained to the patient. All factors, including the amount of tooth structure removal, soft tissue surgery, need for endodontic therapy, expected clinical longevity, and duration of treatment were discussed with the patient and a duly signed consent was obtained.

In the first phase of treatment, oral prophylaxis was performed followed by a thorough root planing. In the second phase, endodontic treatment was performed on the required teeth.

In the third phase of treatment, the periodontal surgery was designed. Once the case was prepared, the surgery was planned and a gingivectomy procedure (external bevel incision) was done as the length of the clinical crowns of almost all teeth were short, and the length had to be increased.

**Figure 3:**
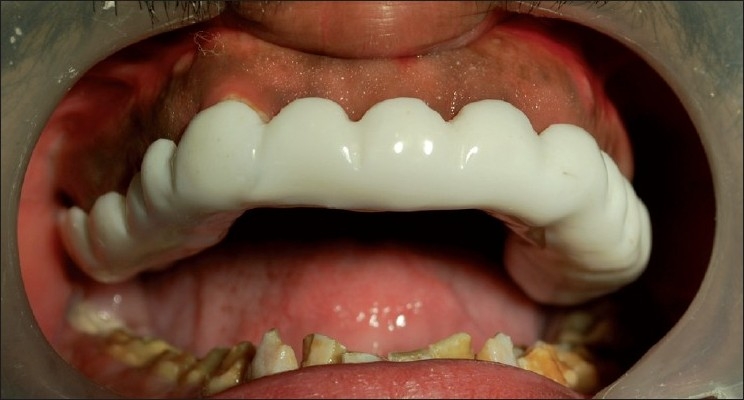
Surgical template inserted in patient’s mouth

**Figure 4:**
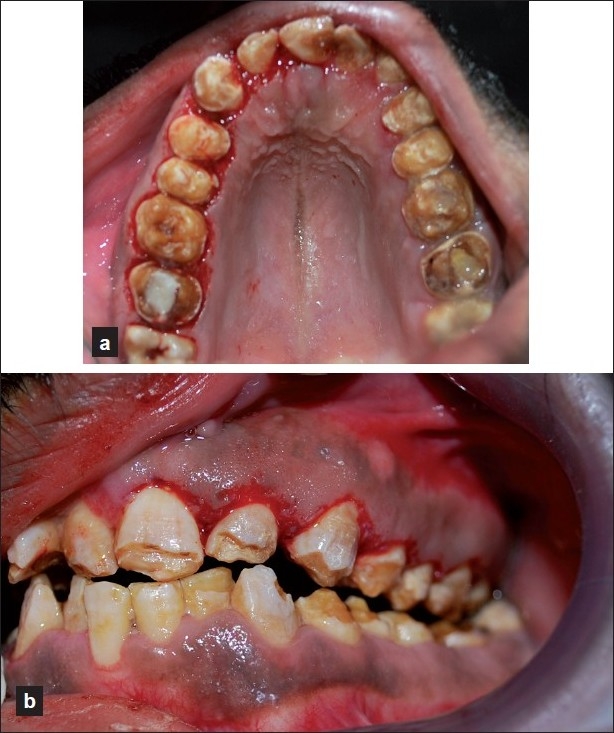
Gingivectomy procedure

Before the surgery, surgical templates were obtained for the patient, from the Department of Prosthodontics, AECS Maaruti College of Dental Sciences and Research Centre, Bangalore. The templates acted as a guide for us to carry out the surgical procedure[8] [Figure 3]. The surgery was planned in such a way that there was no compromise done regarding the biological width. The surgical procedure was completed in a span of 4 weeks [Figure 4].

**Figure 5:**
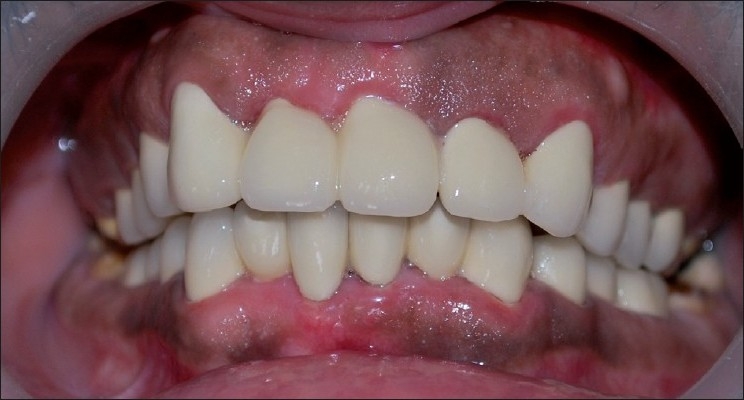
Postoperative view of prosthesis

Once the surgery was completed, the patient was given postsurgical instructions and oral hygiene was reinforced. We waited for about 4–6 weeks for the healing to take place, after which, the patient was referred to the Prosthetic Department, who took care of the crowns, etc [Figure 5].

After the final step, the patient was instructed and counseled to maintain his oral hygiene by proper brushing and dental check up once in at least 6 months.

## DISCUSSION

There are a number of alternatives for the treatment of teeth affected by AI. The treatment of patients with AI presents an interesting challenge to the dentist. The main clinical characteristics are extensive loss of tooth tissue, poor esthetics, and tooth sensitivity. The treatment plan usually varies and many factors have to be taken into consideration–age of the patient, socioeconomic status, and severity of the disorder. The need for crown lengthening is dictated by dental and patient factors. After crown lengthening it should be possible to put restoration margins above, or at, the gingival margin. It is well documented in the literature that this creates a more favorable condition to allow periodontal health. It was found that margins of fixed prosthodontics significantly compromise the gingival health, if placed below the gingival margin.[9] In a study[10] it was found that subgingival margins demonstrated higher plaque, gingival index scores, and probing depths. In addition, when the bacterial morphotypes were examined, there was an increase in the spirochetes, fusiforms, rods, and filamentous bacteria. There is an additional benefit of ease of impression taking, cleansing, and detection of secondary caries. Hence, periodontal health is the cornerstone of any successful restorative procedure. Therefore, the correct handling of the periodontal tissues during restoration of the tooth is important to the restoration’s future success.[11] Thus, it is necessary to prepare periodontal tissues properly before restorative treatment to ensure good form, function, and esthetics of masticatory apparatus and patient comfort. In time bad quality restorations alters the periodontal tissues. Precision of restorations as well as relationship with the periodontium is important. Sometimes even precise restoration can induce inflammation of the periodontal tissue. It is important to know what gingival biological width is, what happens when it is altered, what is lengthening of the clinical crown, and when it should be done.[12]

In order to avoid pathologic changes and to predict treatment results more precisely, it is necessary to keep gingival biological width unaltered during teeth restoration.[13] If there is less than 2 mm distance from restoration’s margin to marginal bone clinical crown lengthening possibility should be considered in dental treatment plan. The choice depends on the relationship of crown–root–alveolar bone and esthetic expectations. The clinical tooth crown could be lengthened surgically or by combining the methods of orthodontic eruption and surgery.

This clinical report describes a treatment sequence based on an interdisciplinary approach. The treatment is usually combined to meet the biological, restorative, and esthetic requirements imposed by short clinical crowns.[14] This technique is generally used to improve esthetics and takes the form of a gingivectomy, to excise the soft tissue.[15] Normally, the gingival margin is 1 mm coronal to the cementoenamel junction. If it is greater, then the clinical crown is shorter than the anatomical crown. In thin tissue biotypes, a gingivectomy will expose more of the crown and improve the appearance. It may be achieved with a scalpel, or with the use of electrosurgery.

## SUMMARY

The complexity of the management of patients with AI supports the suggestion that the dental profession should have appropriate methods for the rehabilitation of rare dental disorders. The treatment of patients with AI should start with early diagnosis and intervention to prevent later restorative problems. This case report describes the functional and esthetic rehabilitation of AI with porcelain-fused-to-metal crowns.
